# Comprehensive subgroup analyses of survival outcomes between clear cell renal cell adenocarcinoma and papillary renal cell adenocarcinoma

**DOI:** 10.1002/cam4.3563

**Published:** 2020-11-03

**Authors:** Jingyi Huang, Da Huang, Jiaqi Yan, Tianhe Chen, Yi Gao, Danfeng Xu, Rong Na

**Affiliations:** ^1^ Department of Urology, Ruijin Hospital Shanghai Jiao Tong University School of Medicine Shanghai China

**Keywords:** histology, kidney cancer, renal cell carcinoma, SEER database

## Abstract

To comprehensively compare the survival outcomes of clear cell renal cell carcinoma (ccRCC) and papillary renal cell carcinoma (pRCC), the study cohort included ccRCC and pRCC patients in 2004–2017 from the Surveillance, Epidemiology, and End Results (SEER) database, which comprises 18 registries. Primary outcomes including overall mortality (OM) and cancer‐specific mortality (CSM) were evaluated. Subgroup analyses were conducted for different ages, race, and disease stages. A total of 112,270 cases were eligible for the current analysis, including 92,209 cases of ccRCC and 20,061 cases of pRCC. Univariate analyses suggested that pRCC has a more favorable outcome than ccRCC in terms of CSM (HR: 0.72, 95% CI: 0.68–0.75, *p* < 0.001) and OM (HR: 0.90, 95% CI: 0.88–0.93, *p* < 0.001). Multivariate‐adjusted HRs suggested that pRCC has worse survival outcomes than ccRCC (adjusted HR: 1.08 for CSM and 1.05 for OM, both *p* < 0.05). Subgroup analyses showed that pRCC had a significantly poorer prognosis than ccRCC among patients ≤45 years old (HR_CSM_: 1.59, 95% CI: 1.31–1.93, *p* < 0.001; HR_OM_: 1.63, 95% CI: 1.40–1.90, *p* < 0.001). Among patients with distant metastasis, those with pRCC had a higher risk of CSM and OM than those with ccRCC (HR_CSM_: 1.28, 95% CI: 1.19–1.39, *p* < 0.001; HR_OM_: 1.30, 95% CI: 1.21–1.40, *p* < 0.001). Propensity score analyses for patients ≤45 years old and those with metastasis showed similar results. The lack of information on pRCC subtypes in the SEER database was a limitation. In conclusion, pRCC has poorer survival outcomes than ccRCC among patients younger than 45 years old and patients with distant metastasis.

## Graphical abstract

Prognosis of pRCC is commonly more favorable than that of ccRCC. But, for patients less than 45 years old and patients with metastatic RCC, the survival outcome of pRCC is worse contrarily.

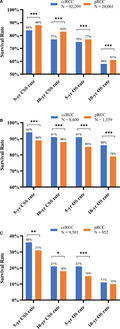

## 1 INTRODUCTION

Renal cell carcinoma (RCC) is the most common type of malignancy in the kidney. It may also be classified into different subtypes, including clear cell renal cell carcinoma (ccRCC) and nonclear cell renal cell carcinoma. Among them, ccRCC accounts for approximately 70% of all diagnosed RCC. Meanwhile, papillary renal cell carcinoma (pRCC) accounts for 10%–15% of RCC and is the most common type of nonclear cell renal cell carcinoma.^1^, ^2^, ^3^ pRCC is considered a relatively indolent subtype. According to the Clinical Guidelines on Renal Cell Carcinoma, patients with pRCC have a better prognosis than those with ccRCC.^4^, ^5^

Recent studies suggested that different outcomes might be observed between pRCC and ccRCC among patients with varying baseline criteria. For example, some studies concluded that the prognosis of localized pRCC was more favorable than that of ccRCC, while the prognosis of advanced/metastatic pRCC was worse than that of ccRCC.^6^, ^7^, ^8^ However, conflicting results were observed in other studies, in which metastatic pRCC and metastatic ccRCC had similar prognoses.^9^, ^10^

In addition to the current contradictory evidence, few studies have focused on comprehensive subgroup analyses based on demographic and clinical factors. In the present study, our purpose was to evaluate the survival outcomes of pRCC and ccRCC using the Surveillance, Epidemiology, and End Results (SEER) database. We intended to investigate whether the outcomes varied among subgroup with different baseline demographic and clinical factors.

## 2 PATIENTS AND METHODS

### 2.1 Study population

The SEER data were obtained from 18 registry research databases using SEER*STAT 8.3.6. The database covers nearly 30% of the total population of the United States.^11^ Patients with either ccRCC (International Classification of Disease for Oncology [ICD‐O‐3] code 8310/3) or pRCC (code 8260/3) from 2004 to 2017 were included in the present study. The exclusion criteria were as follows: 1) unknown survival duration; and 2) uncertain cause of death.

### 2.2 Variables

From the SEER database, we determined the following items as covariables: patients' demographic characteristics (age, race/origin, sex, and age at diagnosis), laterality of the tumor, sequence number, grade of differentiation, stage, presence or absence of bone/brain/liver/lung metastases, and method of surgery. Among them, we categorized age into four groups, ≤45 years, 45–59 years, 60–75 years and ≥75 years. Race/origin was divided into non‐Hispanic white (NHW), non‐Hispanic black (NHB), other non‐Hispanic (ONH, including non‐Hispanic American Indian/Alaska native and non‐Hispanic Asian or Pacific Islander), and Hispanic. Additionally, the type of treatment was classified as no surgery, local tumor excision/destruction, partial nephrectomy, and radical nephrectomy. Cancer‐specific survival (CSS) and overall survival (OS) were regarded as the primary endpoint in our study.

### 2.3 Statistical analysis

Descriptive statistics are applied to illustrate the baseline characteristics. The chi‐squared test was used to compare the categorical variables between the two groups. Furthermore, *t*‐tests were applied to compare normally distributed continuous variables. Non‐normally distributed continuous variables were tested using nonparametric methods. The log‐rank test (Kaplan‐Meier analysis) and univariable Cox hazard regression were used for survival analyses to estimate crude hazard ratios (HRs), and 95% confidence intervals (95% CIs) for overall mortality (OM) and cancer‐specific mortality (CSM). The 5‐year and 10‐year survival rates were compared using the proportion test. Multivariable Cox hazard regression was used to adjust covariates in survival analyses of OM and CSM.

Statistical analyses were implemented with IBM SPSS Statistics for Windows, Version 24.0 (IBM Corp., Armonk, NY, USA). A two‐tailed *p* value of 0.05 or less was considered statistically significant.

## 3 RESULTS

**FIGURE 1:**
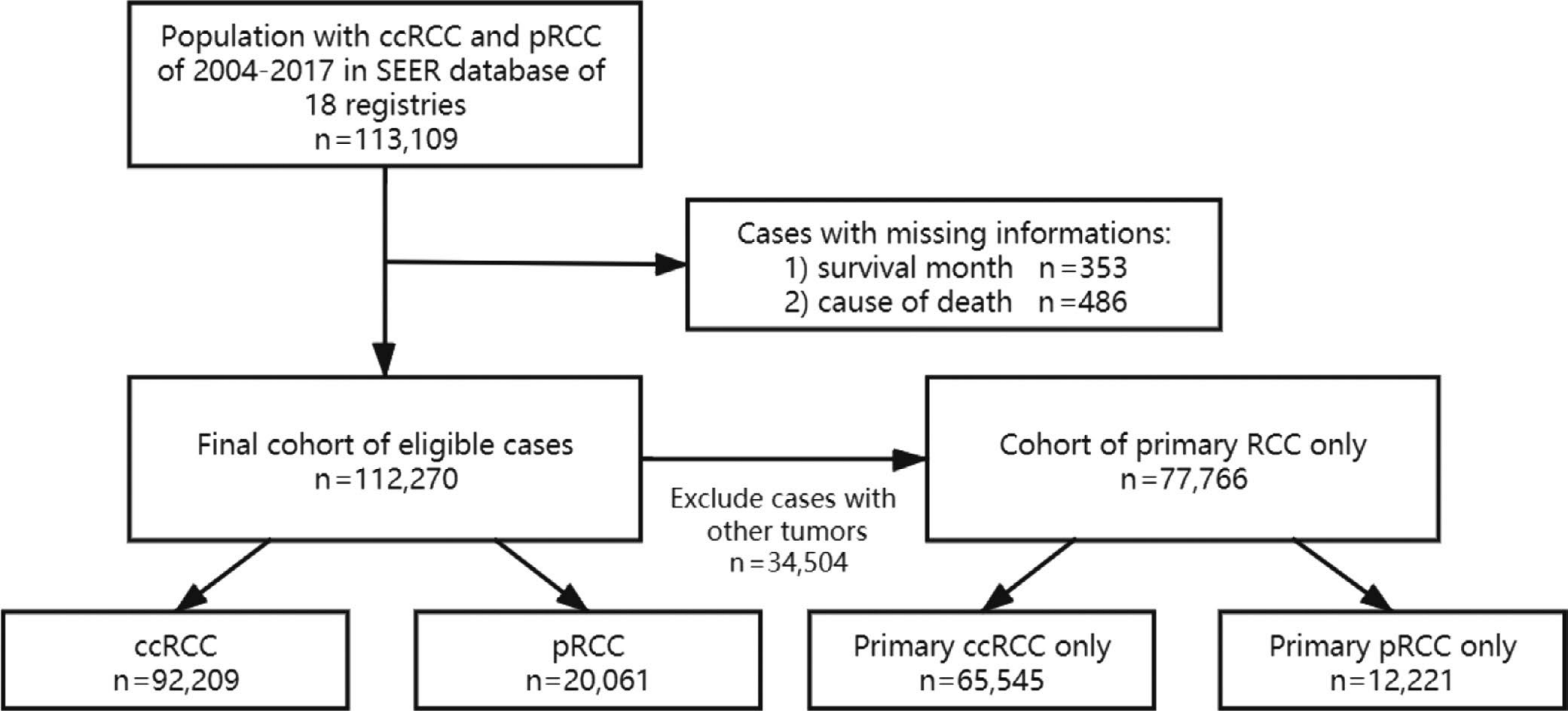
Flowchart for screening eligible cases from SEER database of 18 registries. Abbreviations: ccRCC, clear cell renal cell carcinoma; pRCC, papillary renal cell carcinoma; RCC, renal cell carcinoma.

A total of 113,109 cases were extracted from the SEER database. Figure 1 shows the flowchart of case selection based on the inclusion and exclusion criteria. Finally, 112,270 cases were eligible for further analysis, including 92,209 cases of ccRCC and 20,061 cases of pRCC. Among them, 77,766 cases were of primary RCC only. The median follow‐up time was 46 months (interquartile range, IQR, 18–87 months) for ccRCC and 48 months (IQR, 19–89 months) for pRCC.

Descriptive analyses of the baseline characteristics (including the demographic and clinical characteristics) are presented in Table 1. Comparing the differences in each variable between ccRCC and pRCC, we found that the proportions of male patients (76.9% vs. 62.3%, *p* < 0.001) and NHB patients (28.0% vs. 7.1%, *p* = 0.006) were significantly higher in pRCC than in ccRCC. The age distribution between ccRCC and pRCC was significantly different (*p* < 0.001), with a younger age at diagnosis for ccRCC than for pRCC. Fewer cases of distant metastasis were observed for pRCC than for ccRCC (4.8% vs. 10.4%, *p* < 0.001). In addition, significantly higher CSM (11.1% vs. 15.1%, *p* < 0.001) and OM (25.0% vs. 27.0%, *p* < 0.001) rates were observed for ccRCC than for pRCC.

**TABLE 1:** Characteristics of the study population from the SEER 18 registries research database, 2004–2017.

Characteristics	Entire cohort (n = 112,270)		
	ccRCC (n = 92,209)	pRCC (n = 20,061)	*p* value
Age, n (%)			<0.001
≤45 years	8,600 (9.3)	1,359 (6.8)	
45–59 years	30,098 (32.6)	6,103 (30.4)	
60–74 years	39,186 (42.5)	9,333 (46.5)	
≥75 years	14,325 (15.5)	3,266 (16.3)	
Race/Origin, n (%)			0.006
NHW	64,187 (69.9)	12,577 (63.0)	
NHB	6,553 (7.1)	5,587 (28.0)	
ONH	6,302 (6.9)	645 (3.2)	
Hispanic	14,754 (16.1)	1,151 (5.8)	
Sex, n (%)			<0.001
Male	57,447 (62.3)	15,420 (76.9)	
Female	34,762 (37.7)	4,641 (23.1)	
Grade, n (%)			0.989
1	10,126 (12.8)	1,949 (12.5)	
2	42,385 (53.4)	8,179 (52.5)	
3	21,591 (27.2)	4,922 (31.6)	
4	5,252 (6.6)	528 (3.4)	
Laterality, n (%)			<0.001
Right	46,798 (50.9)	9,954 (49.8)	
Left	44,995 (49.0)	10,001 (50.0)	
Bilateral	85 (0.1)	31 (0.2)	
Stage, n (%)			<0.001
Localized Only	66,695 (72.9)	16,836 (85.0)	
Direct Extension	14,195 (15.5)	1,564 (7.9)	
Nodes Metastasis	1,071 (1.2)	453 (2.3)	
Distant Metastasis	9,501 (10.4)	952 (4.8)	
Surgery, n (%)			<0.001
No Surgery	6,980 (7.6)	1,381 (6.9)	
Radical Nephrectomy	53,717 (58.4)	9,174 (45.9)	
Partial Nephrectomy	27,095 (29.4)	8,073 (40.4)	
Local Tumor Excision	4,215 (4.6)	1,365 (6.8)	
Primary Tumor Only, n (%)	65,545 (71.1)	12,221 (60.9)	<0.001
Bone Metastasis, n (%)	2,320 (3.9)	207 (1.6)	<0.001
Lung Metastasis, n (%)	3,696 (6.1)	292 (2.2)	<0.001
Brain Metastasis, n (%)	712 (1.2)	40 (0.3)	<0.001
Liver Metastasis, n (%)	943 (1.6)	112 (0.8)	<0.001
CSM, n (%)	13,954 (15.1)	2,222 (11.1)	<0.001
OM, n (%)	24,862 (27.0)	5,018 (25.0)	<0.001
OS, [Median (IQR)]	46 (18–87)	48 (19–89)	<0.001

Abbreviations: ccRCC, clear cell renal cell carcinoma; CSS, cancer‐specific survival; IQR, interquartile range; NHB, non‐Hispanic black; NHW, non‐Hispanic white; ONH, other non‐Hispanic; OS, overall survival; pRCC, papillary renal.

**FIGURE 2:**
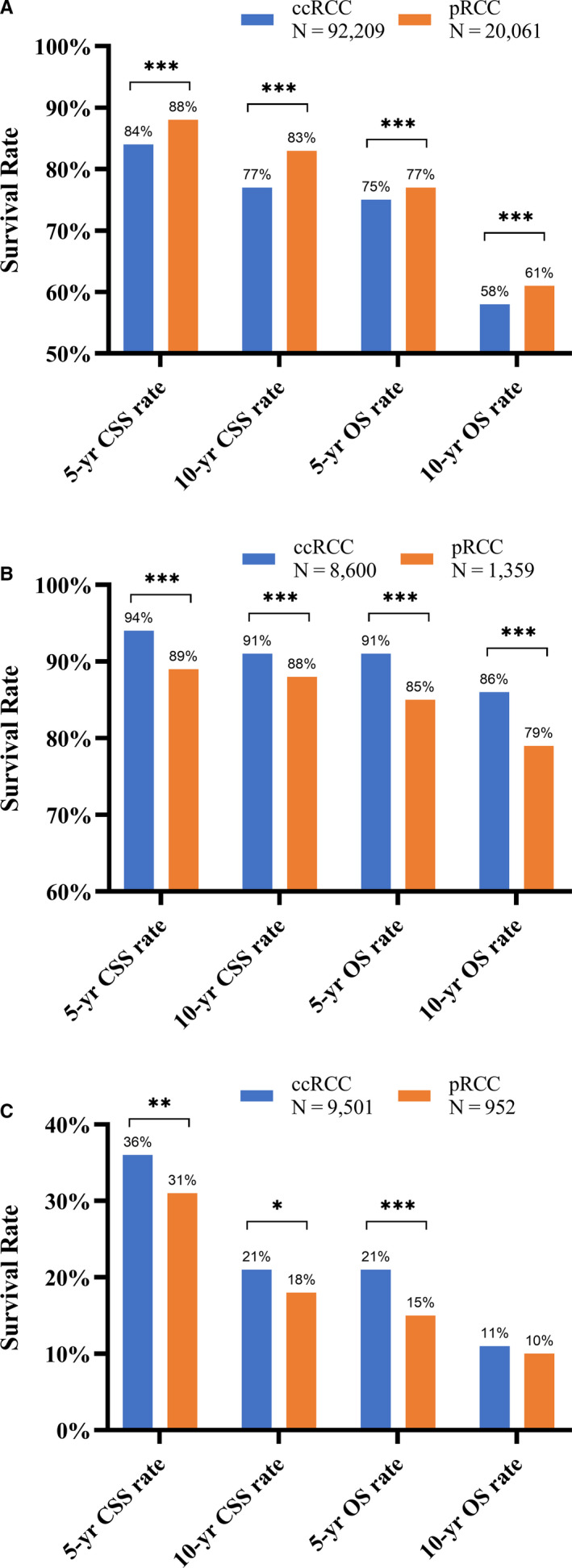
Survival rates by years (5 years and 10 years) of CSS and OS of ccRCC and pRCC (A) in the entire cohort; (B) in the subgroups of ≤45 years; (C) in the subgroups of RCC with distant metastasis. Abbreviations: ccRCC, clear cell renal cell carcinoma; CSS, cancer‐specific survival; OS, overall survival; pRCC, papillary renal; RCC, renal cell carcinoma; ***p < 0.001; **p < 0.01; *p < 0.05.

Table 2 shows the univariate and multivariate analyses of the associations between variables and survival in the entire cohort. In univariate analysis, pRCC had better survival in terms of both CSS (crude HR = 0.72, 95% CI: 0.68–0.75, *p* < 0.001) and OS (HR = 0.90, 95% CI: 0.88–0.93, *p* < 0.001) than ccRCC. We also observed better 5‐year/10‐year survival rates for pRCC (Figure 2A). However, multivariate analysis indicated poorer survival for pRCC (HR_CSS_ = 1.08, R_OS_ = 1.05, both *p* < 0.05) after adjusting for age, sex, race, stage, grade, primary tumor, and surgery method.

**TABLE 2:** Univariable and multivariable cox regression predicting CSS and OS in the entire cohort.

Characteristics	Cancer‐specific survival				Overall survival			
	Crude HR (95% CI)	*p* value	Adjusted HR (95% CI)	*p* value	Crude HR (95% CI)	*P* Value	Adjusted HR (95% CI)	*p* value
Histology								
ccRCC	1.00 (ref.)	‐	1.00 (ref.)	‐	1.00 (ref.)	‐	1.00 (ref.)	‐
pRCC	0.72 (0.68–0.75)	<0.001	1.08 (1.02–1.14)	0.008	0.90 (0.88–0.93)	<0.001	1.05 (1.01–1.09)	0.019
Age								
≤45 years	1.00 (ref.)	‐	1.00 (ref.)	‐	1.00 (ref.)	‐	1.00 (ref.)	‐
45–59 years	1.97 (1.81–2.14)	<0.001	1.40 (1.27–1.54)	<0.001	2.01 (1.88–2.14)	<0.001	1.62 (1.50–1.75)	<.001
60–74 years	2.61 (2.41–2.83)	<0.001	1.82 (1.66–1.99)	<0.001	3.27 (3.07–3.49)	<0.001	2.57 (2.39–2.77)	<.001
≥75 years	4.09 (3.76–4.44)	<0.001	2.77 (2.52–3.06)	<0.001	6.26 (5.86–6.69)	<0.001	4.81 (4.47–5.19)	<.001
Race								
NHW	1.00 (ref.)	‐	1.00 (ref.)	‐	1.00 (ref.)	‐	1.00 (ref.)	‐
NHB	0.88 (0.83–0.92)	<0.001	1.07 (1.01–1.15)	0.036	1.02 (0.98–1.06)	0.356	1.19 (1.14–1.24)	<0.001
ONH	0.96 (0.90–1.03)	0.261	0.96 (0.89–1.03)	0.270	0.87 (0.82–0.91)	<0.001	0.90 (0.85–0.95)	<0.001
Hispanic	0.97 (0.93–1.02)	0.201	1.02 (0.96–1.07)	0.573	0.87 (0.84–0.90)	<0.001	0.97 (0.93–1.01)	0.120
Sex								
Male	1.00 (ref.)	‐	1.00 (ref.)	‐	1.00 (ref.)	‐	1.00 (ref.)	‐
Female	0.83 (0.90–0.85)	<0.001	0.96 (0.92–0.10)	0.041	0.85 (0.83–0.87)	<0.001	0.92 (0.90–0.94)	<0.001
Stage								
Localized only	1.00 (ref.)	<0.001	1.00 (ref.)	<0.001	1.00 (ref.)	<0.001	1.00 (ref.)	‐
Direct extension	3.78 (3.61–3.95)	<0.001	2.65 (2.52–2.79)	<0.001	1.99 (1.93–2.05)	<0.001	1.57 (1.51–1.62)	<0.001
Nodes metastasis	13.83 (12.80–14.94)	<0.001	7.62 (6.98–8.32)	<0.001	5.73 (5.36–6.13)	<0.001	3.92 (3.92–3.63)	<0.001
Distant metastasis	28.08 (27.04–29.15)	<0.001	12.54 (11.93–13.20)	<0.001	10.77 (10.47–11.07)	<0.001	6.15 (5.92–6.39)	<0.001
Surgery								
No surgery	1.00 (ref.)	‐	1.00 (ref.)	‐	1.00 (ref.)	‐	1.00 (ref.)	‐
Radical nephrectomy	0.14 (0.14–0.15)	<0.001	0.29 (0.27–0.31)	<0.001	0.18 (0.174–0.185)	<0.001	0.34 (0.32–0.36)	<0.001
Partial Nephrectomy	0.03 (0.03–0.03)	<0.001	0.12 (0.11–0.14)	<0.001	0.07 (0.07–0.07)	<0.001	0.19 (0.18–0.20)	<0.001
Local tumor excision/destruction	0.06 (0.06–0.07)	<0.001	1.08 (1.03–1.12)	<0.001	0.16 (0.15–0.17)	<0.001	0.37 (0.34–0.40)	<0.001
Primary								
Primary tumor only	1.00 (ref.)	‐	1.00 (ref.)	‐	1.00 (ref.)	‐	1.00 (ref.)	‐
Not only tumor	1.31 (1.26–1.35)	<0.001	1.08 (1.03–1.12)	<0.001	0.80 (0.78–0.81)	<0.001	0.79 (0.77–0.81)	<0.001
Grade	2.47 (2.42–2.53)	<0.001	< 0.001	<0.001	1.65 (1.62–1.67)	<0.001	1.312 (1.289–1.336)	<0.001

Abbreviations: ccRCC, clear cell renal cell carcinoma; HR, hazard ratio; NHB, non‐Hispanic black; NHW, non‐Hispanic white; ONH, other non‐Hispanic; pRCC, papillary renal.

Subgroup analyses of different age groups were also performed (Table 3). Notably, among patients ≤45 years old, significantly poorer survival was observed for pRCC, with an HR = 1.59 (95% CI: 1.31–1.93, *p* < 0.001) for CSS and HR = 1.63 (95% CI: 1.40–1.90, *p* < 0.001) for OS. After adjusting for the above factors in the multivariable analysis, significantly poorer OS was still observed for patients with pRCC (HR = 1.25, 95% CI: 1.02–1.52, *p* = 0.03). We confirmed this result in terms of 5‐year/10‐year survival among patients ≤45 years old as shown in Figure 2B. These results indicated that pRCC has poorer survival than ccRCC among young patients. We performed additional subgroup univariate and multivariate analyses (Table S1). The results showed that the prognosis of pRCC was significantly worse for female patients aged ≤45 years (adjusted HR_CSM_ = 1.56, 95% CI: 1.02–2.40, *p* = 0.042; adjusted HR_OM_ = 1.85, 95% CI: 1.32–2.61, *p* < 0.001). However, such a difference was not observed in young male patients (adjusted HR_CSM_ = 1.06, 95% CI: 0.78–1.44, *p* = 0.042; adjusted HR_OM_ = 1.02, 95% CI: 0.80–1.30, *p* = 0.877).

**TABLE 3:** Univariable and multivariable cox regression predicting CSS and OS with pRCC and ccRCC in subgroups of age.

Characteristics	Cancer‐specific survival				Overall survival			
	Crude HR (95% CI)	*p* value	Adjusted HR (95% CI)	*p* value	Crude HR (95% CI)	*p* value	Adjusted HR (95% CI)	*p* value
≤45 years								
ccRCC	1.00 (ref.)	‐	1.00 (ref.)	‐	1.00 (ref.)	‐	1.00 (ref.)	‐
pRCC	1.59 (1.31–1.93)	<0.001	1.23 (0.97–1.58)	0.092	1.63 (1.40–1.90)	<0.001	1.25 (1.02–1.52)	0.028
45–59 years								
ccRCC	1.00 (ref.)	‐	1.00 (ref.)	‐	1.00 (ref.)	‐	1.00 (ref.)	‐
pRCC	0.66 (0.60–0.72)	<0.001	1.04 (0.92–1.17)	0.530	0.89 (0.83–0.95)	<0.001	1.06 (0.97–1,15)	0.200
60–74 years								
ccRCC	1.00 (ref.)	‐	1.00 (ref.)	‐	1.00 (ref.)	‐	1.00 (ref.)	‐
pRCC	0.63 (0.58–0.67)	<0.001	1.08 (0.99–1.17)	0.089	0.81 (0.77–0.84)	<0.001	1.01 (0.96–1.07)	0.610
≥75 years								
ccRCC	1.00 (ref.)	‐	1.00 (ref.)	‐	1.00 (ref.)	‐	1.00 (ref.)	‐
pRCC	0.78 (0.72–0.85)	<0.001	1.03 (0.92–1.15)	0.650	0.91 (0.86–0.96)	0.001	1.02 (0.96–1.10)	0.518

Abbreviations: 95% CI, 95% confidence interval; ccRCC, clear cell renal cell carcinoma; CSS, cancer‐specific survival; HR, hazard ratio; OS, overall survival; pRCC, papillary renal cell carcinoma.

Due to the different distribution of localized and metastatic diseases between pRCC and ccRCC, we further performed subgroup analyses among patients initially diagnosed with localized RCC versus locally advanced RCC versus metastatic RCC. As shown in Table 4, no significant differences in survival were observed between pRCC and ccRCC among patients with localized or locally advanced diseases (as indicated as “Direct Extension” and “Nodes Metastasis” in Table 4). However, among patients with distant metastatic disease, pRCC had significantly poorer CSS (HR = 1.28, 95% CI: 1.19–1.39, *p* < 0.001) and OS (HR = 1.30, 95% CI: 1.21–1.40, *p* < 0.001) than ccRCC. This effect remained significant after multivariate analysis. Similar results were observed in terms of 5‐year or 10‐year survival rates among metastatic RCCs (Figure 2C).

**TABLE 4:** Univariable and multivariable cox regression predicting CSS and OS with pRCC and ccRCC in subgroups of stage.

Characteristics	Cancer‐specific survival				Overall survival			
	Crude HR (95% CI)	*p* value	Adjusted HR (95% CI)	*p* value	Crude HR (95% CI)	*p* value	Adjusted HR (95% CI)	*p* value
Localized only								
ccRCC	1.00 (ref.)	‐	1.00 (ref.)	‐	1.00 (ref.)	‐	1.00 (ref.)	‐
pRCC	0.98 (0.91–1.05)	0.550	0.95 (0.87–1.03)	0.201	1.10 (1.06–1.14)	<0.001	0.99 (0.94–1.03)	0.517
Direct extension								
ccRCC	1.00 (ref.)	‐	1.00 (ref.)	‐	1.00 (ref.)	‐	1.00 (ref.)	‐
pRCC	0.84 (0.74–0.95)	0.007	0.95 (0.82–1.09)	0.446	0.95 (0.87–1.05)	0.307	1.00 (0.90–1.11)	0.927
Nodes metastasis								
ccRCC	1.00 (ref.)	‐	1.00 (ref.)	‐	1.00 (ref.)	‐	1.00 (ref.)	‐
pRCC	1.04 (0.89–1.21)	0.625	1.11 (0.93–1.33)	0.255	1.08 (0.94–1.24)	0.289	1.11 (0.95–1.31)	0.196
Distant metastasis								
ccRCC	1.00 (ref.)	‐	1.00 (ref.)	‐	1.00 (ref.)	‐	1.00 (ref.)	‐
pRCC	1.28 (1.19–1.39)	<0.001	1.36 (1.21–1.53)	<0.001	1.30 (1.21–1.40)	<0.001	1.35 (1.21–1.51)	<0.001
Non‐advanced								
ccRCC	1.00 (ref.)	‐	1.00 (ref.)	‐	1.00 (ref.)	‐	1.00 (ref.)	‐
pRCC	0.80 (0.75–0.85)	<0.001	0.95 (0.89–1.02)	0.184	1.00 (0.97–1.04)	0.952	0.99 (0.95–1.03)	0.567
Advanced								
ccRCC	1.00 (ref.)	‐	1.00 (ref.)	‐	1.00 (ref.)	‐	1.00 (ref.)	‐
pRCC	1.03 (0.96–1.10)	0.486	1.28 (1.16–1.41)	<0.001	1.06 (1.00–1.13)	0.058	1.27 (1.16–1.40)	<0.001

Abbreviations: 95% CI, 95% confidence interval; ccRCC, clear cell renal cell carcinoma; CSS, cancer‐specific survival; HR, hazard ratio; OS, overall survival; pRCC, papillary renal.

"Non‐Advanced" includes "Localized Only" and "Direct Extension"; "Advanced" includes "Nodes Metastasis" and "Distant Metastasis".

**FIGURE 3:**
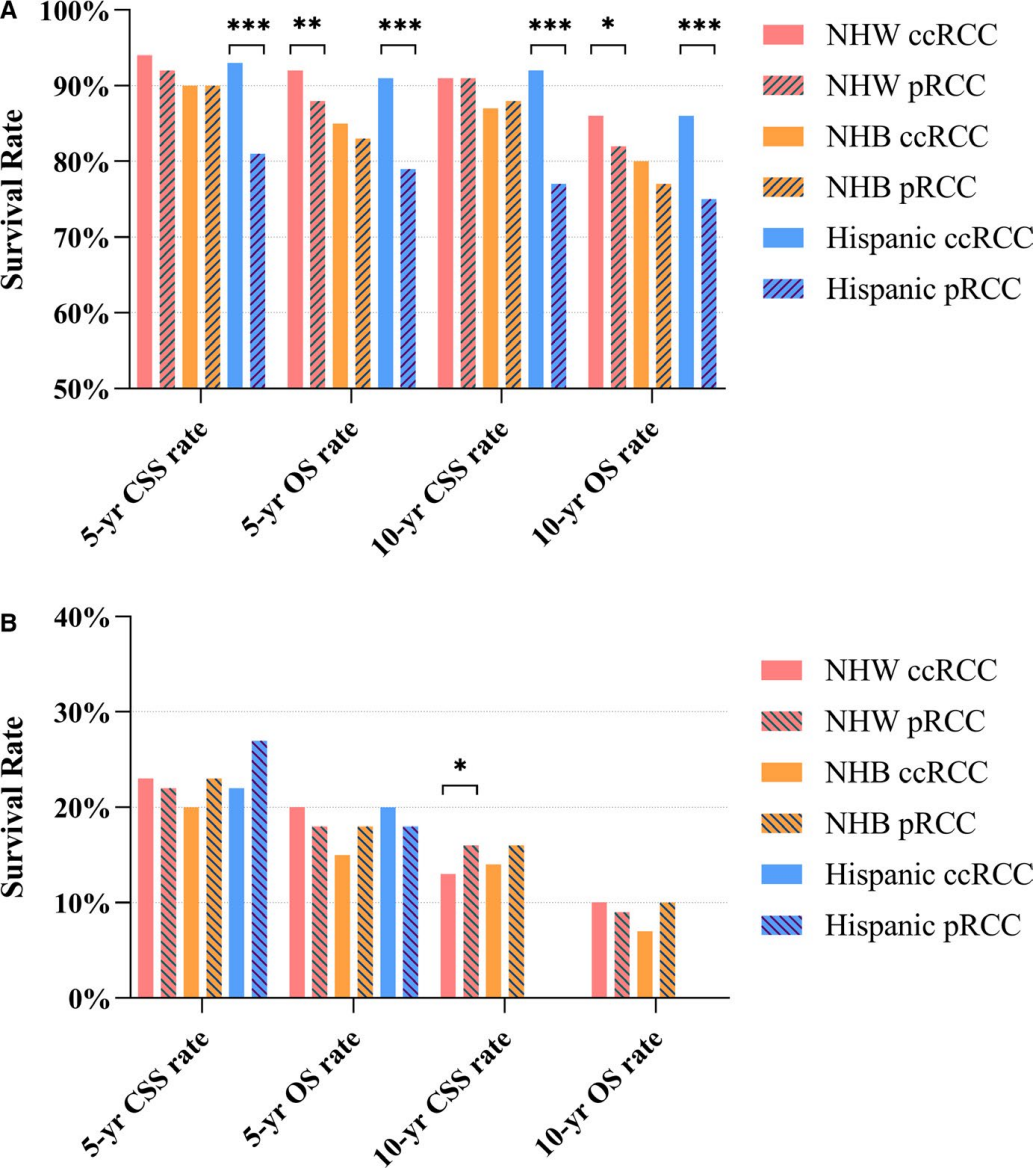
Survival rates by years (5 years and 10 years) of CSS and OS of ccRCC and pRCC for patients of NHW, NHB and Hispanic (A) in the subgroups of ≤45 years; (B) in the subgroups of advanced RCC (as defined as “nodes metastasis” and “distant metastasis”). 10‐years survival rates of RCC in subgroups of Hispanic were not available. Abbreviation: ccRCC, clear cell renal cell carcinoma; CSS, cancer‐specific survival; OS, overall survival; pRCC, papillary renal; RCC, renal cell carcinoma; ***p < 0.001; **p < 0.01; *p < 0.05.

Since there were significant differences in survival between NHB and NHW patients (Table 2), we then investigated whether race would be a potential confounder or effect modifier for survival of RCC. We divided the study cohort into four subgroups based on different ethnicities: NHW, NHB, ONH, and Hispanic. As shown in Table S2, the results were similar to those in the entire cohort. Additional analyses of various age groups and stage groups were also performed among patients with different ethnicities. Notably, a difference in 5‐year/10‐year survival rates between pRCC and ccRCC among patients aged ≤45 years was not observed in NHB patients (Figure 3A). No significant differences in 5‐year/10‐year survival rates were observed between localized and metastatic diseases among different ethnicities (Figure 3B), probably due to the relatively small sample size of each subgroup.

To further investigate whether age, stage of disease, and sex were confounders of survival in RCC, propensity score matching (PSM) was applied with covariables of age, stage, and sex. After PSM, there were 10,389 pairs of completely matched cases and 9,416 pairs after fuzzy matching, including 91,462 cases of ccRCC and 19,805 cases of pRCC. In the propensity score‐matched cohort, pRCC had better survival than ccRCC after adjusting for multiple variables by Cox hazard regression. The adjusted HR for CSM was 0.72 (95% CI: 0.66–0.77; *p* < 0.001) and that for OM was 0.62 (95% CI: 0.59–0.66; *p* < 0.001). Before PSM, there was a significant association between age and disease stage (nonlocalized disease, including direct extension, node metastasis, and distant metastasis patients) among patients aged ≤45 years (16.5% in pRCC vs. 13.5% in ccRCC, *p* = 0.003). Therefore, differences in survival might be driven by the different proportions of nonlocalized disease. After PSM, using the covariates of sex, stage, and race, a total of 3,165 cases of ccRCC and 1,065 cases of pRCC were matched in this subgroup (≤45 years old). Among them, 14.3% of ccRCCs and 15.4% of pRCCs were nonlocalized diseases (*p* = 0.399). The difference was eliminated after PSM. Similar results suggested that pRCC had a poorer prognosis than ccRCC in the ≤45 years subgroup in terms of OS (crude HR_OM_ = 1.35, 95% CI: 1.11–1.64, *p* = 0.003; adjusted HR_OM_ = 1.24, 95% CI: 1.02–1.53, *p* = 0.034).

In consideration of the effect of secondary tumors, all analyses were performed among patients with primary RCCs and without secondary tumors (34,504 patients were excluded, leaving 77,766 cases). The results (Table S3) were consistent with the current results.

Finally, due to the relatively short median follow‐up period (median follow‐up of 46 months for ccRCC and 48 months for pRCC), we further evaluated the associations among subgroups of patients who were enrolled in the SEER database from 2004 to 2014 to ensure that the majority of the consecutive cases would have ~5 years of follow‐up (if not censored because of a loss to follow‐up). With 14,483 cases of pRCC and 66,725 cases of ccRCC from 2004 to 2014, the median follow‐up times were 71 months for ccRCC and 73 months for pRCC. We observed similar results. Briefly, in the subgroups of patients younger than 45 years old or patients with metastatic RCC, the survival outcomes of pRCC were poorer than those of ccRCC (Table S4).

## 4 DISCUSSION

In the current research, we investigated the differences in outcomes between pRCC and ccRCC patients using the SEER database. One of our major findings was that pRCC might have poorer prognosis in certain subgroups of patients: 1) in patients younger than 45 years old, pRCC had a significantly worse survival outcome than ccRCC, but this outcome was not significant among NHB patients (however, a similar estimation was observed); and 2) for distant metastatic RCCs, the prognosis of pRCC was inferior to that of ccRCC; such findings might influence the current knowledge and clinical risk classification standard of pRCC (known as a more indolent malignancy).

A consensus has been widely accepted that patients with pRCC have better OS and CSS than those with ccRCC. However, the multivariable analyses showed that pRCC acted as a risk factor. To exclude potential confounders, subgroup analyses were performed. Previous studies failed to perform subgroup analyses based on different demographic and clinical risk factors, such as sex, age, and ethnicity.^2^, ^4^ Most of these studies focused on the clinical stage and pathology subgroups, which was important but not sufficient. The results in the present study suggest a different but more personalized interpretation of prognosis based on the individuals’ age, ethnicities, etc. In addition, the SEER database might be one of the best cohorts to answer our study hypotheses, as it has such a large sample size and relatively complete follow‐up data. After verifying the impact of age and stage, the outcome of the multivariable analyses after PSM indicated the protective influence of pRCC on prognosis.

Similar to the reported studies, the present results suggested that pRCC with distant metastasis has a worse outcome than ccRCC. This is probably due to the lack of targeted therapies against advanced pRCC. Despite the variety of different targeted therapies for ccRCC (e.g., anti‐vascular endothelial growth factor receptor therapy, also known as anti‐VEGFR therapy, and mTOR pathway‐targeted therapy), there are currently no phase III clinical trial data on non‐ccRCCs.^12^ VEGFR could be observed to be overexpressed in pRCC tissue, indicating a possible response to anti‐VEGFR therapy; however, the treatment effect on metastatic pRCC is insignificant.^13^ Other evidence indicated that pRCC had a poorer response to the current targeted therapies for RCC than ccRCC.^14^

A previous study showed that the African American population had a higher incidence of pRCC (47.9% of all RCCs) than the non‐African American population (10.3% of total RCC).^15^ As mentioned above, the survival rate of NHB patients younger than 45 years old with pRCC and ccRCC was not significantly different from that of the corresponding patients of other races. Distinctions among subgroups of races, especially between NHB and NHW, were also observed when analyzing the survival outcomes of metastatic RCC. Various social factors, such as financial and insurance status, were suspected to be confounders. For example, due to poor accessibility to medical care, NHB patients with ccRCC might not be able to receive appropriate treatment as well as patients of other ethnicities. However, a comparison of the survival rate between metastatic pRCC and metastatic ccRCC indicates that NHB patients with pRCC had a higher survival rate than NHW patients with pRCC, and NHB patients with ccRCC had a lower survival rate than NHW patients with ccRCC. This finding basically overturned the possibility that social factors were confounding factor. After observing the higher morbidity of pRCC in the NHB population, Sankin et al. assumed that there might be a genomic predisposition for black patients to develop pRCC.^15^ Paulucci et al. demonstrated different immune responses to cancer between black and white patients.^16^ Thus, there exists the possibility that genomic or molecular differences between NHB and NHW patients might cause distinct survival outcomes. This conclusion can inspire further explorations.

Several limitations should be noted. First, there are two subtypes of pRCCs, of which papillary type 2 RCC is depicted to have an inferior prognosis to ccRCC.^17^, ^18^, ^19^ Further analysis based on subtypes of pRCC was not performed because subtype information is not available in the SEER database. Second, social factors were not taken into account. The disparity in race usually results in differences in socioeconomic patterns, which may influence the outcomes. Third, the SEER database includes a large number of patients in the US; however, the sample sizes of some subgroups or numbers of events in some subgroups were relatively small, such as the number of cases and events in non‐NHW subgroups. Prospective cohort studies based on different ethnicities are worth conducting in the future to confirm our findings.

## 5 CONCLUSION

In conclusion, the survival outcomes of pRCC are generally more favorable than those of ccRCC. However, in patients younger than 45 years and patients with distant metastatic RCCs, the prognosis of pRCC is worse.

## CONFLICT OF INTEREST

The authors declare to have no competing interest.

## AUTHOR CONTRIBUTIONS

Danfeng Xu and Rong Na were responsible for the study concept and study design. Jingyi Huang and Da Huang acquired the data. Jingyi Huang, Da Huang, and Rong Na analyzed and interpreted the data. Jingyi Huang, Da Huang, Jiaqi Yan, Tianhe Chen, and Yi Gao drafted the manuscript. Danfeng Xu and Rong Na contributed to the critical revision of the manuscript. Danfeng Xu and Rong Na supervised the study. All authors have read and approved the final manuscript.

## Supporting information

Table S1‐S4

## Data Availability

All the data were extracted from the Surveillance, Epidemiology, and End Results (SEER) database released in November 2019. Qualified researchers may access to information on cancer statistics through the website of SEER database (https://seer.cancer.gov/).
