# Supplementary material for: Comprehensive subgroup analyses of survival outcomes between clear cell renal cell adenocarcinoma and papillary renal cell adenocarcinoma
Source: Cancer Med. 2020 Nov 3;9(24):9409–18. doi: 10.1002/cam4.3563 (PMC7774724; doi:10.1002/cam4.3563)
Supplement: Supplementary file 1 — Table S1‐S4 [file CAM4-9-9409-s001.docx]

Supplementary Tables

Content

[Supplementary Table 1 2](#_Toc53001225)

[Supplementary Table 2 3](#_Toc53001226)

[Supplementary Table 3 4](#_Toc53001227)

[Supplementary Table 4 5](#_Toc53001228)

| Supplementary Table 1**.** Cox Regression Predicting CSS and OS with pRCC and ccRCC in Different Groups of Patients in ≤ 45 years subgroup | | | | | | | | | | |  |
| --- | --- | --- | --- | --- | --- | --- | --- | --- | --- | --- | --- |
| Characteristics | | Cancer-Specific Survival | | | |  | Overall Survival | | | | |
|  |  | Crude HR (95%CI) | *p* value | Adjusted HR (95%CI) | *p* value |  | Crude HR (95%CI) | *p* value | Adjusted HR (95%CI) | *p* value | |
| Male | ccRCC | 1.00 (ref.) | - | 1.00 (ref.) | - |  | 1.00 (ref.) | - | 1.00 (ref.) | - | |
|  | pRCC | 1.18 (0.93-1.50) | 0.163 | 1.06 (0.78-1.44) | 0.709 |  | 1.31 (1.09-1.57) | 0.004 | 1.02 (0.80-1.30) | 0.877 | |
| Female | ccRCC | 1.00 (ref.) | - | 1.00 (ref.) | - |  | 1.00 (ref.) | - | 1.00 (ref.) | - | |
|  | pRCC | 3.00 (2.15-4.20) | <0.001 | 1.56 (1.02-2.40) | 0.042 |  | 2.63 (1.98-3.48) | <0.001 | 1.85 (1.32-2.61) | <0.001 | |
| No Surgery | ccRCC | 1.00 (ref.) | - | 1.00 (ref.) | - |  | 1.00 (ref.) | - | 1.00 (ref.) | - | |
|  | pRCC | 0.76 (0.50-1.15) | 0.194 | 0.95 (0.32-2.86) | 0.926 |  | 0.84 (0.58-1.22) | 0.358 | 0.74 (0.29-1.87) | 0.519 | |
| Radical Nephrectomy | ccRCC | 1.00 (ref.) | - | 1.00 (ref.) | - |  | 1.00 (ref.) | - | 1.00 (ref.) | - | |
|  | pRCC | 1.56 (1.24-1.95) | <0.001 | 1.24 (0.95-1.61) | 0.12 |  | 1.77 (1.48-2.12) | <0.001 | 1.39 (1.12-1.73) | 0.003 | |
| Partial Nephrectomy | ccRCC | 1.00 (ref.) | - | 1.00 (ref.) | - |  | 1.00 (ref.) | - | 1.00 (ref.) | - | |
|  | pRCC | 3.01 (1.30-6.98) | 0.01 | 1.05 (0.32-3.39） | 0.939 |  | 1.00 (0.60-1.66) | 0.985 | 0.63 (0.34-1.17） | 0.145 | |
| Local Tumor Excision  /Destruction | ccRCC | 1.00 (ref.) | - | 1.00 (ref.) | - |  | 1.00 (ref.) | - | 1.00 (ref.) | - | |
|  | pRCC | 2.41 (0.22-26.54) | 0.473 | NA | - |  | 1.48 (0.48-4.53) | 0.497 | 0.65 (0.11-4.08) | 0.65 | |
| Grade 1 | ccRCC | 1.00 (ref.) | - | 1.00 (ref.) | - |  | 1.00 (ref.) | - | 1.00 (ref.) | - | |
|  | pRCC | 0.73 (0.17-3.10) | 0.667 | 0.70 (0.13-3.83) | 0.681 |  | 1.11 (0.57-2.15) | 0.759 | 0.70 (0.32-1.52) | 0.363 | |
| Grade 2 | ccRCC | 1.00 (ref.) | - | 1.00 (ref.) | - |  | 1.00 (ref.) | - | 1.00 (ref.) | - | |
|  | pRCC | 1.74 (1.06-2.86) | 0.030 | 1.44 (0.81-2.57) | 0.220 |  | 1,67 (1.22-2.30) | 0.002 | 1.40 (0.98-2.01) | 0.064 | |
| Grade 3 | ccRCC | 1.00 (ref.) | - | 1.00 (ref.) | - |  | 1.00 (ref.) | - | 1.00 (ref.) | - | |
|  | pRCC | 1.40 (1.04-1.90) | 0.029 | 1.41 (0.99-2.00) | 0.055 |  | 1.45 (1.11-1.88) | 0.006 | 1.37 (1.02-1.85) | 0.037 | |
| Grade 4 | ccRCC | 1.00 (ref.) | - | 1.00 (ref.) | - |  | 1.00 (ref.) | - | 1.00 (ref.) | - | |
|  | pRCC | 1.05 (0.66-1.66) | 0.852 | 0.91 (0.55-1.51) | 0.712 |  | 1.08 (0.69-1.68) | 0.736 | 0.99 (0.61-1.61) | 0.965 | |

Abbreviation: HR, hazard ratio; ccRCC, clear cell renal cell carcinoma; pRCC, papillary renal; mRCC, metastatic renal cell carcinoma; CSS, cancer-specific survival; OS, overall survival

| Supplementary Table 2**.** Univariable and Multivariable Cox Regression Predicting CSS and OS with pRCC and ccRCC in Subgroups of Race | | | | | | | | | | |
| --- | --- | --- | --- | --- | --- | --- | --- | --- | --- | --- |
| Characteristics | | Cancer-Specific Survival | | | |  | Overall Survival | | | |
|  |  | Crude HR (95%CI) | *p* value | Adjusted HR (95%CI) | *p* value |  | Crude HR (95%CI) | *p* value | Adjusted HR (95%CI) | *p* value |
| NHW | ccRCC | 1.00 (ref.) | - | 1.00 (ref.) | - |  | 1.00 (ref.) | - | 1.00 (ref.) | - |
|  | pRCC | 0.69 (0.66-0.73) | < 0.001 | 1.09 (1.01-1.16) | 0.020 |  | 0.87 (0.84-0.90) | < 0.001 | 1.03 (0.99-1.08) | 0.192 |
| NHB | ccRCC | 1.00 (ref.) | - | 1.00 (ref.) | - |  | 1.00 (ref.) | - | 1.00 (ref.) | - |
|  | pRCC | 0.71 (0.64-0.79) | < 0.001 | 1.04 (0.91-1.19) | 0.567 |  | 0.86 (0.81-0.93) | < 0.001 | 1.06 (0.97-1.15) | 0.197 |
| ONH | ccRCC | 1.00 (ref.) | - | 1.00 (ref.) | - |  | 1.00 (ref.) | - | 1.00 (ref.) | - |
|  | pRCC | 1.000 (0.803-1.245) | 0.997 | 1.39 (1.06-1.82) | 0.016 |  | 1.00 (0.85-1.19) | 0.975 | 1.24 (1.01-.152) | 0.039 |
| Hispanic | ccRCC | 1.00 (ref.) | - | 1.00 (ref.) | - |  | 1.00 (ref.) | - | 1.00 (ref.) | - |
|  | pRCC | 0.83 (0.70-1.00) | 0.046 | 1.05 (0.85-1.31) | 0.640 |  | 0.94 (0.82-1.07) | 0.316 | 1.02 (0.87-1.20) | 0.774 |

Abbreviation: HR, hazard ratio; ccRCC, clear cell renal cell carcinoma; pRCC, papillary renal; mRCC, metastatic renal cell carcinoma; CSS, cancer-specific survival; OS, overall survival; NHW, non-Hispanic white; NHB, non-Hispanic black; ONH, other non-Hispanic

| Supplementary Table 3**.** Cox Regression Predicting CSS and OS with pRCC and ccRCC in Patients with Primary RCC Only | | | | | | | | | | |
| --- | --- | --- | --- | --- | --- | --- | --- | --- | --- | --- |
| Characteristics | | Cancer-Specific Survival | | | |  | Overall Survival | | | |
|  |  | Crude HR (95%CI) | *p* value | Adjusted HR (95%CI) | *p* value |  | Crude HR (95%CI) | *p* value | Adjusted HR (95%CI) | *p* value |
| Entire | ccRCC | 1.00 (ref.) | - | 1.00 (ref.) | - |  | 1.00 (ref.) | - | 1.00 (ref.) | - |
|  | pRCC | 0.72 (0.68-0.76) | <0.001 | 1.11 (1.03-1.19) | <0.001 |  | 0.87 (0.84-0.91) | <0.001 | 1.08 (1.03-1.14) | 0.003 |
| ≤ 45 years | ccRCC | 1.00 (ref.) | - | 1.00 (ref.) | - |  | 1.00 (ref.) | - | 1.00 (ref.) | - |
|  | pRCC | 1.74 (1.42-2.14) | <0.001 | 1.22 (0.94-1.59) | 0.131 |  | 1.77 (1.49-2.10) | <0.001 | 1.31 (1.06-1.63) | 0.014 |
| Distant Metastasis | ccRCC | 1.00 (ref.) | - | 1.00 (ref.) | - |  | 1.00 (ref.) | - | 1.00 (ref.) | - |
|  | pRCC | 1.33 (1.22-1.45) | <0.001 | 1.39 (1.22-1.58) | <0.001 |  | 1.32 (1.21-1.43) | <0.001 | 1.37 (1.21-1.55) | <0.001 |

Abbreviation: HR, hazard ratio; ccRCC, clear cell renal cell carcinoma; pRCC, papillary renal; mRCC, metastatic renal cell carcinoma; CSS, cancer-specific survival; OS, overall survival

| Supplementary Table 4**.** Cox Regression Predicting CSS and OS with pRCC and ccRCC in Patients Diagnosed from 2004 to 2014 | | | | | | | | | | |
| --- | --- | --- | --- | --- | --- | --- | --- | --- | --- | --- |
| Characteristics | | Cancer-Specific Survival | | | |  | Overall Survival | | | |
|  |  | Crude HR (95%CI) | *p* value | Adjusted HR (95%CI) | *p* value |  | Crude HR (95%CI) | *p* value | Adjusted HR (95%CI) | *p* value |
| Entire | ccRCC | 1.00 (ref.) | - | 1.00 (ref.) | - |  | 1.00 (ref.) | - | 1.00 (ref.) | - |
|  | pRCC | 0.71 (0.67-0.74) | < 0.001 | 1.05 (0.99-1.12) | 0.088 |  | 0.90 (0.87-0.93) | < 0.001 | 1.03 (1.00-1.08) | 0.092 |
| ≤ 45 years | ccRCC | 1.00 (ref.) | - | 1.00 (ref.) | - |  | 1.00 (ref.) | - | 1.00 (ref.) | - |
|  | pRCC | 1.55 (1.26-1.90) | < 0.001 | 1.20 (0.93-1.56) | 0.163 |  | 1.59 (1.36-1.87) | < 0.001 | 1.23 (1.00-1.51) | 0.051 |
| Distant Metastasis | ccRCC | 1.00 (ref.) | - | 1.00 (ref.) | - |  | 1.00 (ref.) | - | 1.00 (ref.) | - |
|  | pRCC | 1.22 (1.12-1.33) | < 0.001 | 1.30 (1.15-1.48) | < 0.001 |  | 1.22 (1.11-1.35) | < 0.001 | 1.29 (1.14-1.45) | <0.001 |

Abbreviation: HR, hazard ratio; ccRCC, clear cell renal cell carcinoma; pRCC, papillary renal; mRCC, metastatic renal cell carcinoma; CSS, cancer-specific survival; OS, overall survival
